# Differentiating prostate cancer from benign prostatic hyperplasia using whole-lesion histogram and texture analysis of diffusion- and T2-weighted imaging

**DOI:** 10.1186/s40644-021-00423-5

**Published:** 2021-09-27

**Authors:** Pengyi Xing, Luguang Chen, Qingsong Yang, Tao Song, Chao Ma, Robert Grimm, Caixia Fu, Tiegong Wang, Wenjia Peng, Jianping Lu

**Affiliations:** 1 Department of Radiology, Changhai Hospital of Shanghai, The Second Military Medical University, No.168 Changhai Road, 200433 Shanghai, China; 2 Application Predevelopment, Siemens Healthcare, Erlangen, Germany; 3 MR Application Development, Siemens Shenzhen Magnetic Resonance Ltd, Shenzhen, China

**Keywords:** Prostate cancer, Prostatic Hyperplasia, Magnetic resonance imaging, Diffusion

## Abstract

**Background:**

To explore the usefulness of analyzing histograms and textures of apparent diffusion coefficient (ADC) maps and T2-weighted (T2W) images to differentiate prostatic cancer (PCa) from benign prostatic hyperplasia (BPH) using histopathology as the reference.

**Methods:**

Ninety patients with PCa and 112 patients with BPH were included in this retrospective study. Differences in whole-lesion histograms and texture parameters of ADC maps and T2W images between PCa and BPH patients were evaluated using the independent samples t-test. The diagnostic performance of ADC maps and T2W images in being able to differentiate PCa from BPH was assessed using receiver operating characteristic (ROC) curves.

**Results:**

The mean, median, 5^th^, and 95^th^ percentiles of ADC values in images from PCa patients were significantly lower than those from BPH patients (*p* < 0.05). Significant differences were observed in the means, standard deviations, medians, kurtosis, skewness, and 5^th^ percentile values of T2W image between PCa and BPH patients (*p* < 0.05). The ADC_5th_ showed the largest AUC (0.906) with a sensitivity of 83.3 % and specificity of 89.3 %. The diagnostic performance of the T2W image histogram and texture analysis was moderate and had the largest AUC of 0.634 for T2W_Kurtosis_ with a sensitivity and specificity of 48.9% and 79.5 %, respectively. The diagnostic performance of the combined ADC_5th_ & T2W_Kurtosis_ parameters was also similar to that of the ADC_5th_ & ADC_Diff−Variance_.

**Conclusions:**

Histogram and texture parameters derived from the ADC maps and T2W images for entire prostatic lesions could be used as imaging biomarkers to differentiate PCa and BPH biologic characteristics, however, histogram parameters outperformed texture parameters in the diagnostic performance.

## Background

Prostate cancer (PCa) is the second most commonly diagnosed cancer in men worldwide [1]. Patients with suspected PCa usually undergo a standard transrectal ultrasound-guided biopsy. However, increased serum prostate-specific antigen (PSA) levels and abnormal digital rectal examinations have also been used as detection methods. Unfortunately, these tests have resulted in the insufficient detection of high-grade PCa tumors and excessive detection of low-grade lesions [2]. Although a few randomized-controlled trials have shown that patients with low-grade tumors failed to benefit from therapy, most patients continued to have excessive therapeutic interventions and follow-up examinations, which increased patient and the healthcare sector medical costs [3, 4].

With the increasing application of multi-parametric magnetic resonance imaging (mpMRI), more focal PCa tumors can be detected and accurately localized, making early and precise PCa therapies possible [5]. Diffusion-weighted imaging (DWI) is a noninvasive technique used to evaluate the microscopic mobility of water molecules in tissues and has been used to detect and evaluate prostatic tumors [6, 7]. Apparent diffusion coefficient (ADC) maps, derived from DWI images, can reflect the histologic characteristics of lesions and has enhanced PCa diagnosis as a supplementary diagnostic tool [8]. Kuhl et al. found that bi-parametric MRI (bpMRI, T2-weighted imaging, and DWI) and mpMRI (T2WI, DWI, and dynamic contrast-enhanced MRI) had similar diagnostic efficiency and accuracy. bpMRI image interpretations were also have a good consistency among radiologists, and the diagnostic accuracy of tumor detection was similar to that of mpMRI. However, bpMRI had significantly shorter imaging acquisition and interpretation time, and no contrast agent is needed compared with mpMRI [9]. Dynamic contrast-enhanced (DCE) MRI characterizes the pharmacokinetic tissue properties through imaging during the administration of contrast agent. However, this method has several limitations, such as potential adverse reactions to gadolinium administration, additional scanning time and cost, and poor consistency in the interpretation of images among radiologists, limiting its broad application in clinical practice [10, 11].

Image texture analysis can be used to estimate the heterogeneity of image signals by quantifying the roughness and regularity of grayscale pixel value spatial distributions in normal and pathologic tissues; the macroscopic heterogeneity of images might reflect microscopic heterogeneity at the level of histopathology [12, 13]. Several studies have shown that MR image texture analysis could detect, classify, evaluate, and predict breast, brain, rectal, and cervical cancer lesions [14–17]. Sidhu et al. used single-slice texture analysis of ADC, T2W, and contrast-enhanced T1W images to identify clinically significant carcinomas in patients with transitional prostatic lesions [18]. Wibmer et al. found that Haralick texture analysis of prostate MRI could be used to detect PCa and differentiate Gleason scores [19]. Differentiating PCa from benign prostatic hyperplasia (BPH) remains a challenge using conventional multi-parametric MRI due to lesion heterogeneity. Histogram and texture image analysis is a promising tool that provides a numerical representation of data distributions and is particularly useful when evaluating the heterogeneous features of tumors [14, 20, 21]. Cui et al. evaluated the diagnostic performance of histogram analysis of intravoxel incoherent motion parameters for differentiating PCa from BPH [22]. Chatterjee et al. found that ADC values were significantly lower in PCa compared to all BPH types and can differentiate between PCa and BPH with high accuracy (areas under the curve: AUC = 0.87) [23]. Bonekamp et al. compared biparametric contrast-free radiomic machine learning, mean ADC, and radiologist assessment for characterization of prostate lesions detected during prospective MRI interpretation. They reported an AUC of 0.84 for mean ADC values and validated their results with a test cohort of 133 patients [24]. Peng et al. reported AUC values for the differentiation of PCa from normal foci of the 10^th^ percentile ADC, average ADC, T2-weighted skewness, and Ktrans [25]. However, using whole-lesion histogram and texture analysis with bpMRI to distinguish BPH nodules from PCa has not yet been reported.

Therefore, the study aimed to explore the usefulness of ADC map and T2W image histogram and texture analyses to distinguish PCa from BPH using histopathology as the reference.

## Methods

### Subjects

This retrospective study was approved by the local institutional review board (NO. M20140149), and individual consent for this retrospective analysis was waived. Between March 2015 and July 2017, consecutive patients with pathologically proven PCa or BPH were enrolled in this study. Patients who met the following criteria were included in the present study: (1) had pathologically proven prostatic hyperplasia (systemic biopsy) or cancer (prostatectomy); (2) had prostatic MRI examinations performed; (3) the interval between prostatic biopsy/radical resection and MRI was less than 3 months; and (4) no history of other malignant tumors. The exclusion criteria were as follows: (1) histopathology of lesion biopsies were confirmed to be positive but were negative on MRI; (2) pretreatments were given to treat prostatic lesions, such as endocrine, chemotherapy, or radiotherapy; (3) poor image quality due to motion artifacts or severe susceptibility artifacts; and (4) incomplete imaging protocol, images of DWI or T2W were missed to perform histogram and texture analysis. Patient clinical characteristics were recorded, including age, PSA levels, lesion volumes, score of Prostate Imaging Reporting and Data System (PI-RADS, version 2), and Gleason scores (in the case of lesions was confirmed to be PCa).

### Magnetic resonance imaging

All imaging was performed on a 3 Tesla MRI system (MAGNETOM Skyra, Siemens Healthcare, Erlangen, Germany) using a standard 18-channel phased-array body coil and 32-channel integrated spine coil. The main parameters of axial DWI were repetition time/echo time (TR/TE) = 5100/89 ms, field of view (FOV) = 224 × 280 mm^2^, matrix = 120 × 150, slices = 20, slice thickness = 4 mm, gap = 0 mm, acceleration factor = 2, *b*-values (number of averages) = 0 (1), 500(2), 800(4), 1000(5), 1500(6), 2000(8) s/mm^2^, diffusion gradients applied in three orthogonal directions, and acquisition times = 6 min 43 s. ADC maps were inline calculated using the mono-exponential model, *S(b) = S(0) e*^− b*ADC^, where *S(b)* is the signal intensity with a *b*-value > 0, and *S(0)* is the signal intensity with a *b*-value = 0. Parameters for axial T2-weighted turbo spin echo sequence were TR/TE = 5460/104 ms, FOV = 180 × 180 mm^2^, matrix = 384 × 384, slices = 24, slice thickness = 4 mm, gap = 0 mm, echo train length = 18, and acquisition time = 3 min 49 s.

### Image analysis

All images were sent to a dedicated workstation for data processing and were independently assessed by two experienced radiologists (P.X. and Q.Y.) with 6 and 8 years of experience in pelvic radiology, respectively. The radiologists were blinded to the data and clinical information, and using a consensus, selected the largest lesions in patients with multicentric or multifocal tumors for further analysis.

**Fig. 1:**
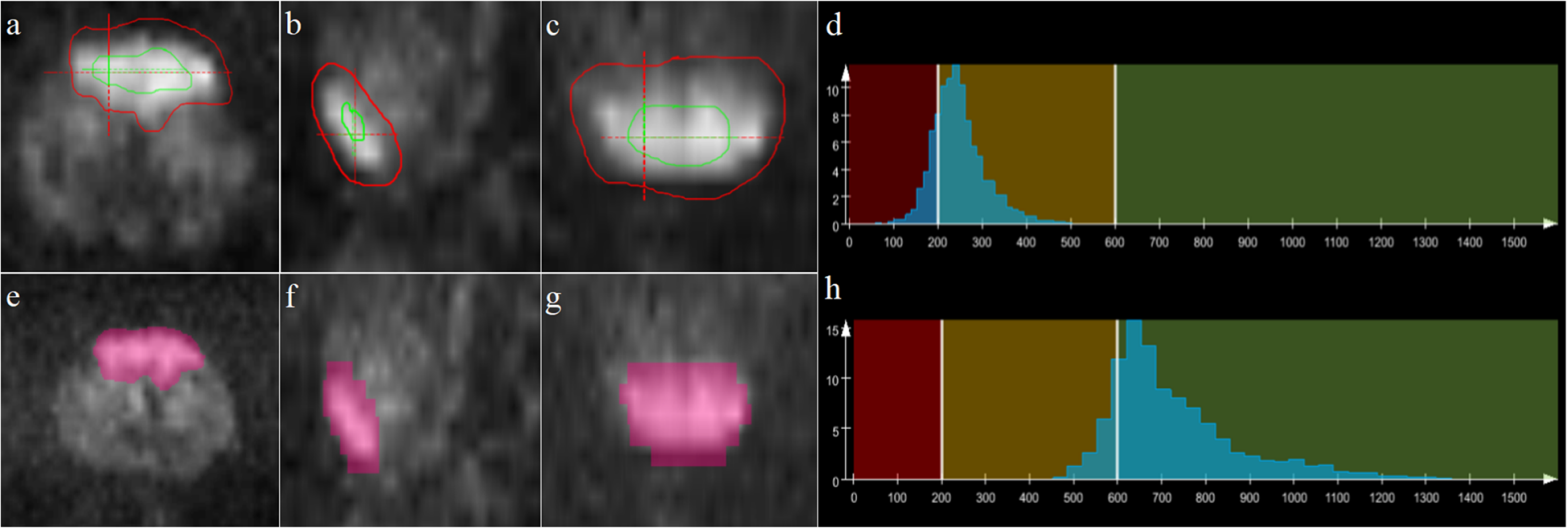
Flow diagrams of the whole-lesion histogram and texture analysis. **a**-**c**: Foreground and background seed points were manually drawn inside (green color) and outside (red color) on three reformatted diffusion-weighted imaging (DWI) images with *b* = 1500 s/mm^2^. **e**-**g**: Three-dimensional segmentations were generated on the DWI images. **d, h**: Histograms of the T2weighted (T2W) images and apparent diffusion coefficient (ADC) maps that were generated

Whole-lesion histogram and texture analyses were performed on ADC maps and T2W images with the prototypic MR Multiparametric Analysis software (Siemens Healthcare, Erlangen, Germany) by the radiologists using the following steps: (1) Import data. T2W images, DWI images with b = 1500 s/mm^2^, and ADC maps were loaded into the histogram and texture analysis software. (2) Region of interest (ROI) delineations were acquired. Foreground and background seed points were manually drawn inside and outside of lesions, respectively, on three reformatted planes of the DWI images. Then, they were automatically copied to ADC maps and T2W images. (3) Lesion segmentation. Segmentation of the whole lesion was performed based on seed points with a random walker algorithm [14]. Manual adjustments for segmentation were performed, if necessary. (4) Histogram and texture analysis. Histogram and texture analyses for entire lesions on the ADC maps and T2W images were performed, and statistical parameters were extracted, including lesion volume, mean, standard deviation, median, 5^th^ and 95^th^ percentiles, differential variance (diff-variance), differential entropy (diff-entropy), contrast, entropy, skewness, and kurtosis. Figure 1 shows the workflow of histogram and texture analysis.

### Histopathologic analysis

All patients underwent transrectal ultrasound-guided biopsy or radical prostatectomy. Biopsies and prostatectomies were formalin-fixed and subjected to tissue sectioning. Tissue sections were stained with a hematoxylin & eosin stain and subjected to immunohistochemical analyses. A urologic pathologist with 8 years of experience, observed the histologic sections and determined lesion locations and boundaries. If the lesions were confirmed to be PCa, Gleason scores were determined according to the PCa grading guidelines formulated by the 2014 Consensus of the International Urological Pathology Association [26]. For patients with BPH, the largest hyperplastic nodules were selected for analysis, while those with PCa had the largest lesions chosen for analysis.

### Statistical analysis

Statistical analyses were performed using SPSS software (Version 19, IBM Statistical Package for the Social Sciences, Chicago, IL) and MedCalc (Version 18.2.1, MedCalc Software, Mariakerke, Belgium). Quantitative variables are presented as the mean ± standard deviation or as the median (1^st^ and 3^rd^ quartile ranges) based on the normal distribution of the data, and the categorical variables are expressed as percentages. Differences in PI-RADS score, PSA levels, and lesion volumes between the PCa and BPH patients were compared using Mann-Whitney U tests. Statistical variable differences in age, histograms and texture analyses of the ADC maps and T2W images between PCa and BPH patients were evaluated using the independent-samples t-test. In addition, the diagnostic performance of histogram and texture parameters on ADC maps and T2W images in being able to differentiate PCa from BPH was assessed using receiver operating characteristic (ROC) curves, and the 95 % confidence interval (CI) for the area can be used to test the hypothesis that the theoretical area is 0.5. If the CI does not include the 0.5 value, then there is evidence that the laboratory test does have an ability to distinguish between the two groups [27, 28]. Furthermore, we explored the diagnostic performance of optimized and combined parameters, based on the best diagnostic performance of the ADC map and/or T2W image histogram and texture parameters. A *p*-value < 0.05 was considered statistically significant.

## Results

### Patients

Two hundred and thirteen patients with prostatic lesions were enrolled in this study. Among these, 11 patients were excluded because three had poor image quality, two had lesions confirmed to be positive by histopathologic diagnoses but had negative MRI results, four had endocrine, chemotherapy, or radiotherapy before MRI, and two had incomplete imaging protocol. Finally, a total of 202 patients with a mean age of 65.9 ± 8.7 years (range 37–86 years) were included in the final analysis. Ninety (44.5 %) patients were diagnosed with PCa, and 112 (55.5 %) were diagnosed with BPH. There were significant differences between PCa and BPH patients in median scores (quartile range) of PI-RADS (5 (4, 5) vs. 2 (2, 2), *p* < 0.0001), age (68.9 ± 7.4 vs. 63.5 ± 8.9 years, *p* < 0.001), PSA concentrations (14.88 (9.05, 30.13) vs. 9.81 (7.26, 15.01) ng/ml, *p* < 0.001), and lesion volumes (2.50 (1.10, 6.00) vs. 1.05 (0.70, 1.70) cm^3^, *p* < 0.001) (Table 1). The number of PCa patients with Gleason scores of 3 + 3, 3 + 4, 4 + 3 and ≥ 8 were 27 (13.4 %), 17 (8.4 %), 14 (6.9 %) and 32 (15.8 %), respectively.

**Table 1:** Patient characteristics

Variable	Value	
	PCa	BPH
Patients (*n*, %)	90 (44.5)	112 (55.5)
Age (years) ^a^	68.9±7.4	63.5±8.9
PSA level (ng/ml) ^b^	14.88(9.05, 30.13)	9.81(7.26, 15.01)
Lesion volume (ml) ^b^	2.50(1.10, 6.00)	1.05(0.70, 1.70)
PI-RADS score ^b^	5 (4, 5)	2 (2, 2)
Gleason score		
3+3 (*n*, %)	27 (13.4)	/
3+4 (*n*, %)	17 (8.4)	/
4+3 (*n*, %)	14 (6.9)	/
≥8 (*n*, %)	32 (15.8)	/

*PCa* prostate cancer, *BPH* benign prostatic hypertrophy, *PSA* prostate-specific antigen, *PI-RADS* prostate imaging reporting and data system

^a^, and ^b^, data are expressed as the mean ± standard deviation and the median (1^st^ and 3^rd^ quartile ranges), respectively

### Comparisons of histogram and texture parameters

The statistical results regarding differences in histogram and texture parameters of ADC maps and T2W images in patients with PCa and BPH are summarized in Table 2. Histogram parameters of the mean, median, and 5^th^ and 95^th^ percentiles of ADC maps were significantly lower in PCa patients compared with those in BPH patients (all *p* < 0.0001). For ADC maps, aside from kurtosis (0.419 ± 1.212 vs. 0.315 ± 1.311, *p* = 0.386), standard deviation, diff-variance, diff-entropy, contrast, entropy, and skewness measures were significantly larger in PCa patients than in BPH patients (all *p* < 0.05). Significant differences in the means, standard deviations, medians, kurtosis and skewness values, and 5^th^ percentiles of T2W images were found between PCa and BPH patients (all *p* < 0.05), while no significant differences were observed in the 5^th^ percentile, diff-variance, diff-entropy, entropy or contrast parameters.

**Table 2:** Histogram parameters of apparent diffusion coefficient (ADC) maps and T2weighted (T2W) images in patients with prostate cancer (PCa) and benign prostatic hyperplasia (BPH)

	Parameters	PCa (*n* = 90)	BPH (*n* = 112)	*p*-value
**ADC**	5^th^ percentile	557.661 ± 131.688	795.973 ± 116.08	< 0.0001
	Mean	806.754 ± 131.268	988.752 ± 106.763	< 0.0001
	Median	788.539 ± 140.309	979.942 ± 111.27	< 0.0001
	Std	176.311 ± 48.874	129.004 ± 45.47	< 0.0001
	Diff-Variance	0.195 ± 0.032	0.178 ± 0.04	< 0.0001
	Diff-Entropy	0.757 ± 0.099	0.707 ± 0.086	< 0.0001
	Contrast	0.628 ± 0.231	0.488 ± 0.21	< 0.0001
	Entropy	1.748 ± 0.289	1.605 ± 0.234	< 0.0001
	95^th^ percentile	1123.756 ± 169.511	1217.362 ± 142.675	< 0.0001
	Skewness	0.472 ± 0.597	0.297 ± 0.575	0.026
	Kurtosis	0.419 ± 1.212	0.315 ± 1.311	0.386
**T2W**	Kurtosis	2.272 ± 2.179	1.375 ± 1.598	0.001
	Skewness	0.851 ± 0.558	0.631 ± 0.413	0.001
	5^th^ percentile	171.594 ± 43.653	193.165 ± 45.757	0.001
	Median	259.822 ± 54.330	278.165 ± 54.817	0.014
	Std	68.67 ± 20.296	61.937 ± 18.164	0.012
	Mean	267.630 ± 56.500	283.756 ± 55.486	0.039
	Entropy	2.355 ± 0.291	2.362 ± 0.279	0.797
	Diff-Variance	0.589 ± 0.178	0.570 ± 0.162	0.578
	95^th^ percentile	391.011 ± 88.876	393.134 ± 78.278	0.759
	Diff-Entropy	1.467 ± 0.158	1.459 ± 0.157	0.808
	Contrast	1.847 ± 0.689	1.851 ± 0.665	0.954

*Std* standard deviation, *Diff-Variance* difference in variance, *Diff-Entropy* difference in entropy

### The diagnostic performance of the histogram and texture parameters

**Fig. 2:**
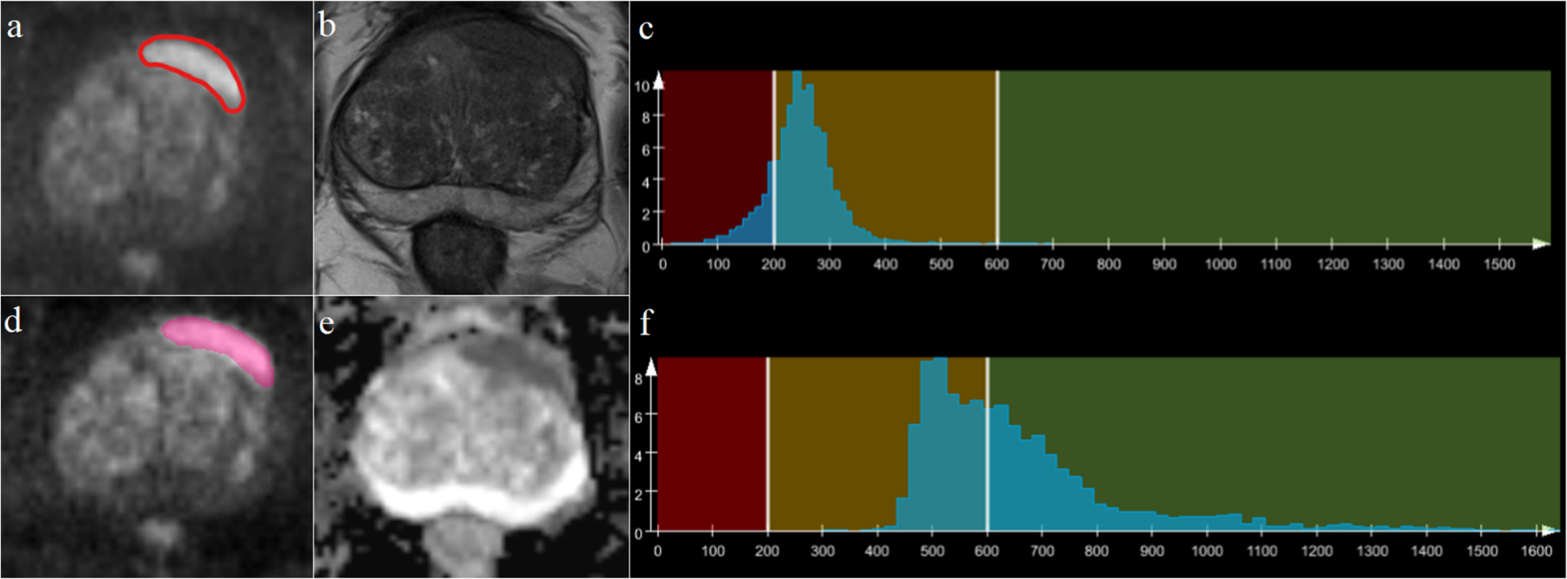
Representative images of a 69-year-old male with prostatic carcinoma (**a-f**) and Gleason scores of 4 + 3. Segmentation of the lesion is shown on diffusion-weighted imaging (DWI) images with a *b* = 1500 s/mm^2^ (**a, d**). Histograms (**c**, **f**) of T2weighted (T2W) images (**b**) and ADC maps (**e**)

**Fig. 3:**
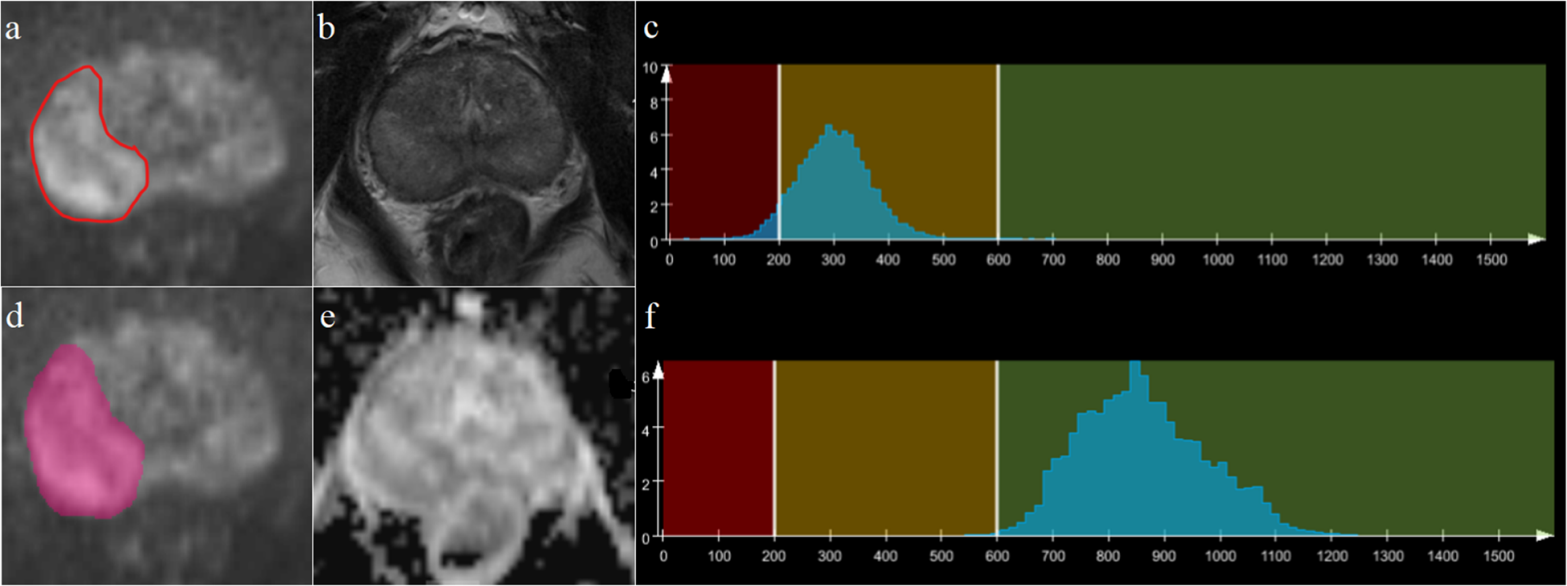
Representative images of a 63-year-old-male patient with benign prostatic hypertrophy (**a-f**). Segmentation of the lesion is shown on diffusion-weighted imaging (DWI) images with a *b* = 1500 s/mm^2^ (**a, d**). Histograms (**c**, **f**) of T2weighted (T2W) images (**b**) and apparent diffusion coefficient (ADC) maps (**e**) were inline-generated

**Fig. 4:**
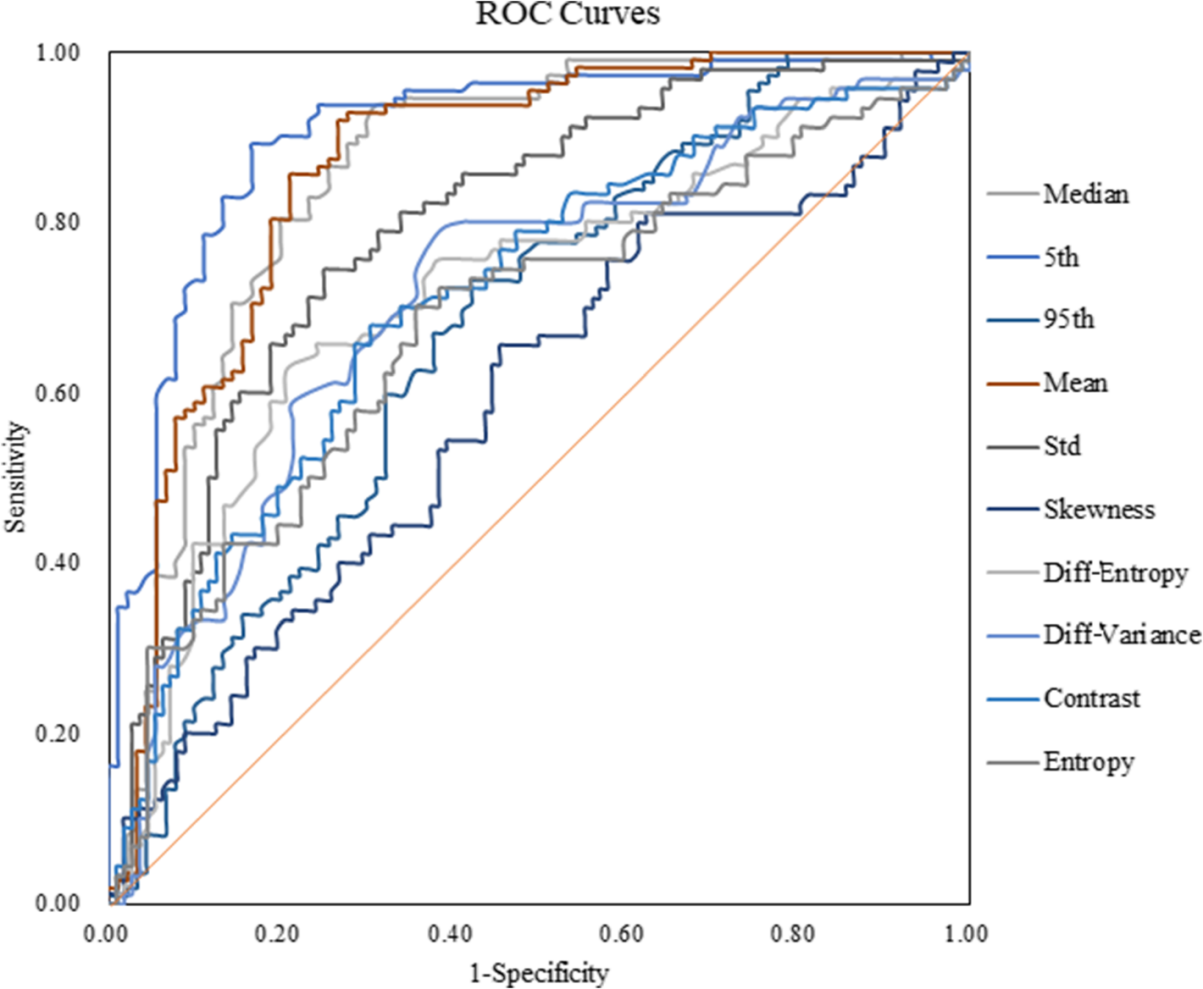
Receiver operating characteristic (ROC) curves show the diagnostic performance in distinguishing prostatic carcinoma from benign prostatic hypertrophy with different ADC map histogram parameters. The 5^th^ percentile of the ADC maps (ADC_5th_) showed the best overall sensitivity and specificity compared with the other parameters

**Fig. 5:**
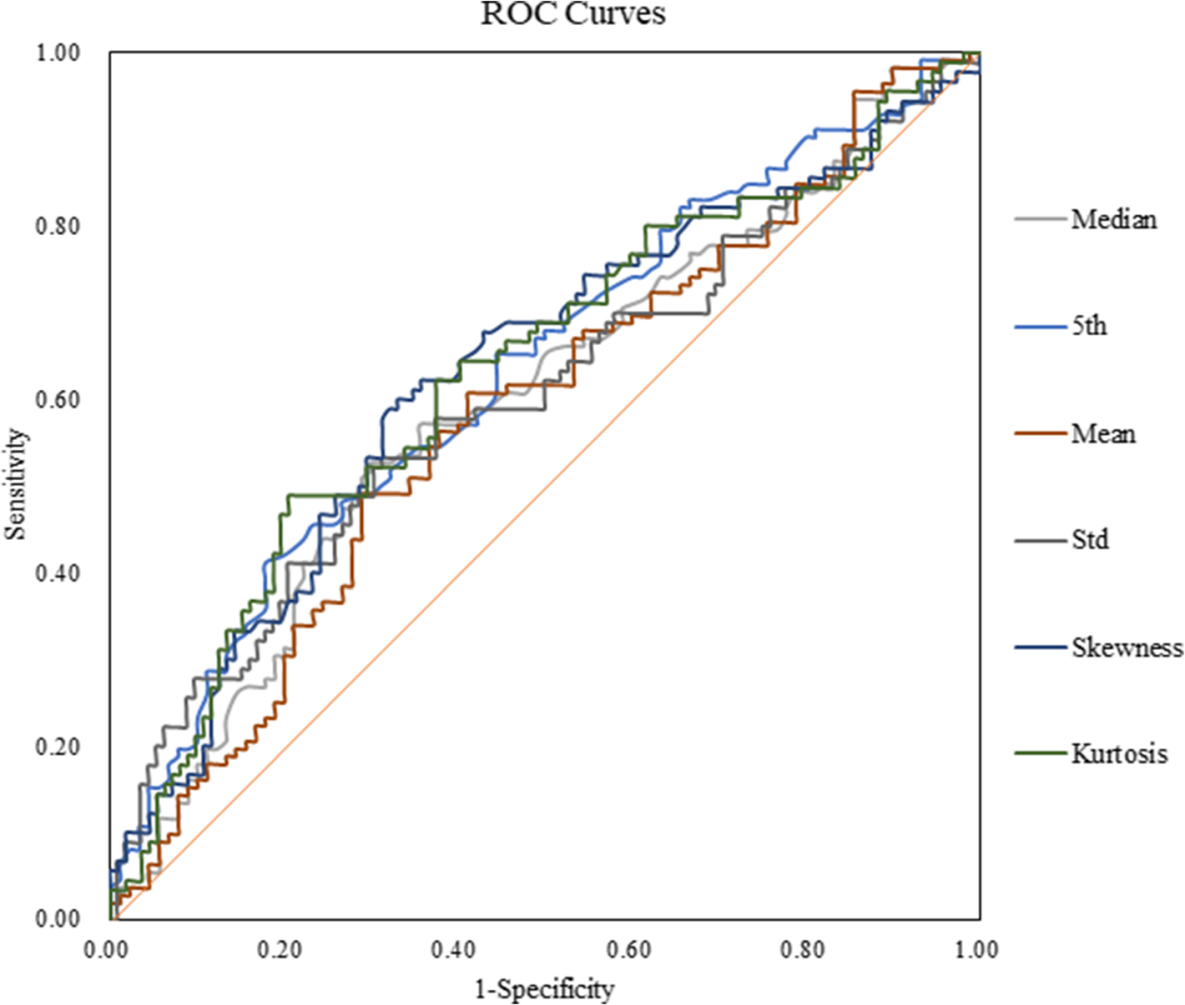
Receiver operating characteristic (ROC) curves show the diagnostic performance in being able to distinguish prostatic carcinoma from benign prostatic with different T2W image histogram parameters

**Fig. 6:**
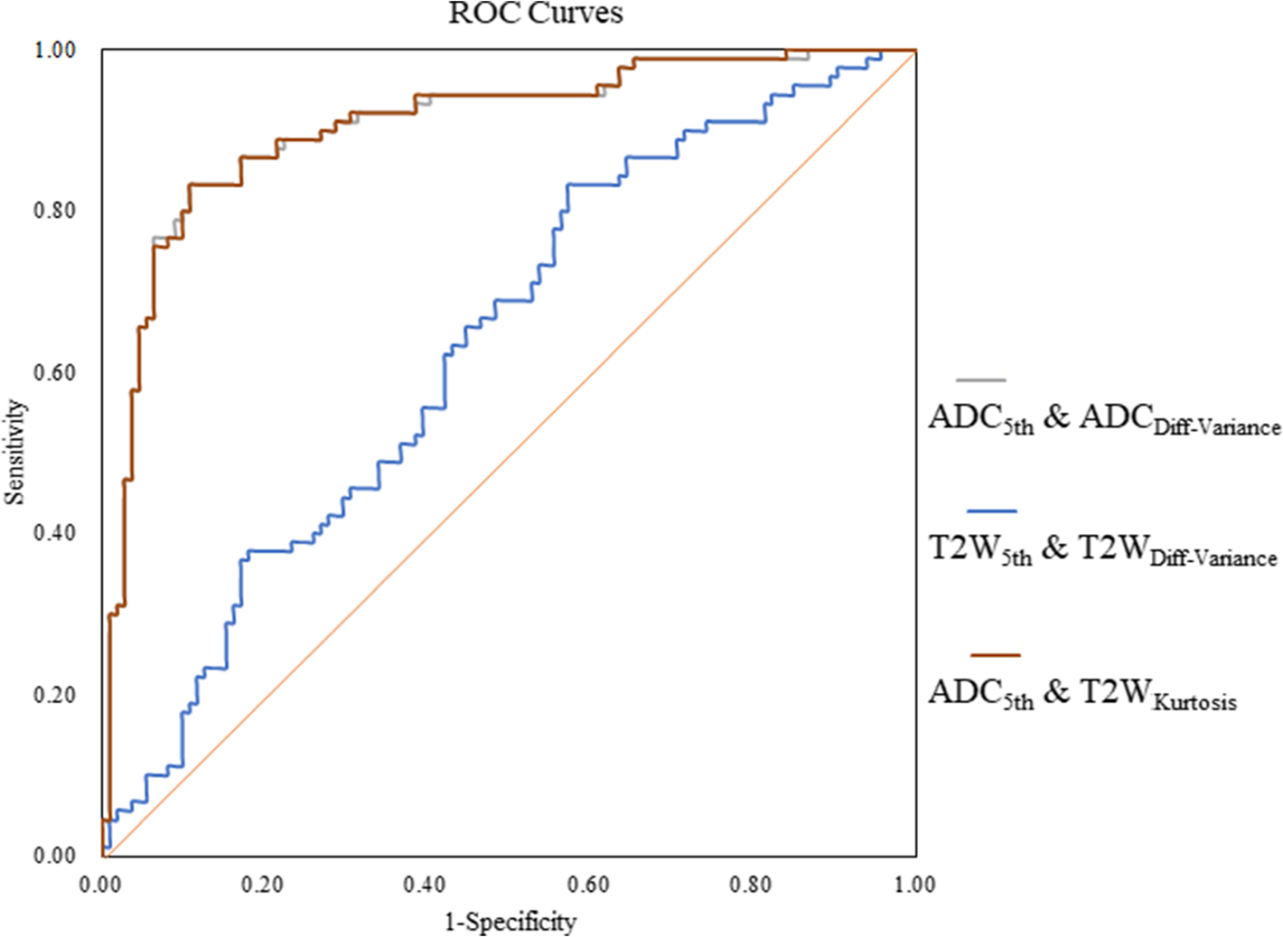
Receiver operating characteristic (ROC) curves show the diagnostic performance in being able to distinguish prostatic carcinoma from benign prostatic with different combined ADC map histogram parameters. This graph shows improved sensitivity and specificity when the 5^th^ percentile of the ADC values (ADC_5th_) and ADC differential variance (ADC_Diff−Variance_) are used together, but this combination is not better than 5^th^ percentile of the ADC maps (ADC_5th_) parameter used alone (Fig. 4)

Table 3 shows that the ROC results of ADC map qualitative histogram and texture analyses were able to differentiate PCa from BPH. The AUCs for the ADC maps ranged from 0.536 to 0.906 with a sensitivity and specificity of 53.3–83.3 % and 57.1–89.3 %, respectively. Moreover, the 5^th^ percentile of the ADC maps showed the largest AUC (0.906) with a sensitivity and specificity of 83.3 and 89.3 %, respectively, while kurtosis had the lowest AUC (0.536) with a sensitivity and specificity of 53.3 and 57.1 %, respectively. Histogram and texture analysis workflow are shown with two representative PCa and BPH patients (Figs. 2 and 3). ROC curve analyses for the histogram and texture parameters of ADC maps and T2W images are shown in Fig. 4. Compared with ADC maps, the diagnostic performance of T2W images in the histogram and texture analyses was moderate, while kurtosis had the largest AUC of 0.634 with a sensitivity and specificity of 48.89% and 79.46 %, respectively (Table 3; Fig. 5). In addition, the diagnostic performance of combining the 5^th^ percentile of the ADC values (ADC_5th_) & the kurtosis of T2W (T2W_Kurtosis_) parameters was the same as that of the combined ADC_5th_ & ADC_Diff−Variance_ parameters, yielding AUCs of 0.906 (95 % CI 0.857, 0.943), and sensitivities and specificities of 83.3 %, and 89.3 %, respectively. However, these combined parameters were not better than the ADC_5th_ parameter alone in distinguishing PCa from BPH, which was also true of the T2W_5th_ & T2W_Diff − Variance_ combined parameters (Table 4; Fig. 6).

**Table 3:** Receiver operating characteristic curve results regarding the qualitative analysis of apparent diffusion coefficient (ADC) maps and T2weighted (T2W) images to distinguish prostate cancer from benign prostatic hyperplasia

	Parameters	AUC (95 % CI)	Sensitivity (%)	Specificity (%)	Cutoff value	*p* value	Youden index	+LR	-LR
**ADC**	5^th^ percentile	0.906 (0.858, 0.943)	83.3	89.3	≤ 650.5	< 0.0001	0.7262	7.78	0.19
	Mean	0.866 (0.811, 0.910)	73.3	92.0	≤ 858.379	< 0.0001	0.653	9.13	0.29
	Median	0.861 (0.805, 0.905)	68.9	93.7	≤ 830.5	< 0.0001	0.6264	11.02	0.33
	Std	0.797 (0.734, 0.850)	74.4	75.0	> 142.175	< 0.0001	0.4944	2.98	0.34
	Diff-Variance	0.717 (0.649, 0.778)	78.9	61.6	> 0.18	< 0.0001	0.405	2.05	0.34
	Diff-Entropy	0.717 (0.650, 0.778)	63.3	78.6	> 0.746	< 0.0001	0.419	2.96	0.47
	Contrast	0.712 (0.644, 0.773)	67.8	69.6	> 0.506	< 0.0001	0.3742	2.23	0.46
	Entropy	0.681 (0.612, 0.744)	70.0	64.3	> 1.668	< 0.0001	0.3429	1.96	0.47
	95^th^ percentile	0.674 (0.605, 0.738)	57.8	73.2	≤ 1135.5	< 0.0001	0.3099	2.16	0.58
	Skewness	0.591 (0.520, 0.660)	65.6	54.5	> 0.299	0.0250	0.2002	1.44	0.63
	Kurtosis	0.536 (0.464, 0.606)	53.3	57.1	> 0.089	0.3910	0.1048	1.24	0.82
**T2W**	Kurtosis	0.634 (0.563, 0.700)	48.89	79.46	> 2.082	0.0008	0.2835	2.38	0.64
	Skewness	0.633 (0.562, 0.699)	60	66.96	> 0.653	0.0009	0.2696	1.82	0.6
	5^th^ percentile	0.633 (0.562, 0.699)	82.22	41.07	≤ 206.5	0.0007	0.2329	1.4	0.43
	Median	0.601 (0.530, 0.669)	70	52.68	≤ 278.5	0.012	0.2268	1.48	0.57
	Std	0.603 (0.532, 0.671)	53.33	68.75	> 66.431	0.0119	0.2208	1.71	0.68
	Mean	0.584 (0.513, 0.653)	71.11	49.11	≤ 288.803	0.0371	0.2022	1.4	0.59
	Entropy	0.511 (0.439, 0.581)	43.33	69.64	≤ 2.29	0.7993	0.1298	1.43	0.81
	Diff-Variance	0.523 (0.452, 0.593)	25.56	82.14	> 0.711	0.5799	0.07698	1.43	0.91
	95^th^ percentile	0.513 (0.441,0.583)	88.89	3.57	≤ 515.5	0.7625	0.0754	0.92	3.11
	Diff-Entropy	0.510 (0.439, 0.581)	42.22	65.18	> 1.526	0.8086	0.07401	1.21	0.89
	Contrast	0.502 (0.431, 0.573)	32.22	74.11	≤ 1.426	0.954	0.0633	1.24	0.91

*AUC* area under the curve, *Std* standard deviation, *Diff-Variance* difference in variance, *Diff-Entropy* difference in entropy

**Table 4:** Receiver operating characteristic curve results regarding the qualitative analysis of the combined parameters to distinguish prostate cancer from benign prostatic hyperplasia

Parameters	AUC (95 % CI)	Sensitivity (%)	Specificity (%)	Cutoff value	*p* value	Youden index	+LR	-LR
ADC_5th_ & T2W_Kurtosis_	0.906 (0.857, 0.943)	83.3	89.3	> 0.53384	< 0.0001	0.7262	7.78	0.19
ADC_5th_ & ADC_Diff−Variance_	0.906 (0.857, 0.942)	83.3	89.3	> 0.53566	< 0.0001	0.7262	7.78	0.19
T2W_5th_ & T2W_Diff − Variance_	0.633 (0.562, 0.699)	83.3	42.9	> 0.38111	0.0007	0.2619	1.46	0.39

*ADC *apparent diffusion coefficient, *T2W* T2-weighted imaging, *5th* 5^th^ percentile, *Diff-variance* difference in variance

## Discussion

In the present study, we evaluated the whole-lesion histogram and texture analyses of ADC maps and T2W images to distinguish PCa and BPH using histopathologic diagnoses as the reference standard and found those parameters could serve as useful biologic characterizations of PCa. The results demonstrated that histogram and texture analyses of parameters from ADC and T2W images could be useful to differentiate PCa from BPH, however, histogram parameters outperformed texture parameters in the diagnostic performance. All histogram and texture parameters, except for kurtosis, were significantly different in ADC values between PCa and BPH patients. Significant differences were observed in the means, standard deviations, medians, kurtosis and skewness values, and the 5^th^ percentile of T2W images between PCa and BPH lesions.

DWI detects the Brownian motion of water molecules and closely reflects tumor microenvironments, such as tumor cell densities, water content, the amount of fibrous stroma, and cell membrane integrities [29]. ADC maps can provide in vivo quantitative diffusion measurements. Several studies have shown that ADC values were negatively correlated with Gleason classifications [30–32]. Absolute ADC values can vary depending on the choice and number of *b* values selected. Thus, the current guidelines do not recommend using a single quantitative ADC parameter to characterize lesions [33]. ADC values have also shown reasonable repeatability in vivo, with a variation of about 20 %. In these studies, evaluations of mean or median ADCs were the primary focus [34, 35]. A few studies have demonstrated the mean relative percentage variations in ADC of prostate ranging from 6.45 to 15.93 % during single scanning session or when two scans were performed within 2 weeks [36, 37].

ADC map histogram and texture parameters showed good diagnostic capabilities in detecting and characterizing diseases and evaluating therapeutic responses. These parameters can determine the spatial variations of ADC values and provide additional information about tumor heterogeneity, which could better reflect tumor characteristics than simply averaging these differences with ROIs. In this study, we found that texture features extracted from ADC maps of prostatic MRI could be used as potential biomarkers to distinguish BPH from PCa tumors. Although this is a preliminary study, the imaging phenotype based on the whole-lesion histograms of MR multi-parametric maps might provide as a noninvasive tool to evaluate the biological characteristics and heterogeneity of PCa.

T2WI has been used to show prostatic zonation anatomies to localize lesions, which has served as the key protocol for prostatic MRI since it was first described in the early 1980 s [38]. Tan et al. performed a meta-analysis and reported that the overall sensitivity and specificity of PCa detection using T2WI were 0.57–0.62 and 0.74–0.78, respectively [39]. Due to its low diagnostic efficacy, T2WI should not be used alone in clinical practice. In thePI-RADS version 2, T2WI is involved in prostatic lesion scoring, but overall lesions are judged primarily based on DWI [40]. This finding is consistent with the differing abilities of T2WI and DWI texture analyses in being able to detect PCa and BPH in our study. Downes et al. created a unique histologic sub-pattern using the standard Gleason grading system, where T2WI was used to evaluate potential histopathologic differences between interstitial and epithelial tumor components [41]. Nketiah et al. showed that T2W image texture features were more sensitive than signal intensities in revealing tissue morphologies and were closely related to potential pathophysiologic changes in PCa tumors, further improving the existing PCa classification methods [42]. Daniel et al. showed that bpMRI texture analysis could distinguish normal tissues from tumor tissues in patients with androgen deprivation therapy better compared with traditional histogram parameters [43]. Our study revealed that whole-lesion histogram and texture analysis parameters of T2W images could be used to distinguish PCa from BPH; however, the diagnostic performance was low compared with those of ADC maps.

Texture analysis has been used to diagnose, differentiate, and assesses the types and therapeutic effects of various tumors, including PCa. Studies have shown that histogram and texture analyses of ADCs contributed to the characterization of prostate tumors. Compared with the mean and median ADC values and 90^th^ percentile Kapp values of diffusion kurtosis imaging, the 10^th^ percentile ADC values correlated better with Gleason classifications, and was superior to other DWI parameters in being able to distinguish low-grade and high-grade tumors [31, 44].

Limited whole-lesion histogram analysis has been used to evaluate PCa therapeutic responses [45]. Kyriazi et al. proved that the 25^th^ percentiles of ADC were the best predictor of chemotherapeutic responses in patients with metastatic ovarian cancer and primary peritoneal cancers [46]. Xie et al. used the histogram and texture analyses of ADCs to differentiate triple-negative breast cancers from other subtypes [14]. Another group used DCE MRI and texture analysis to differentiate malignant glioma from glioblastoma [17]. Texture analysis parameters, extracted from T2W images of rectal cancer patients, were also found to be useful imaging biomarkers to assess tumor responses to neoadjuvant chemotherapies [16]. Meng et al. revealed possible T2W and ADC texture parameters that could be used as noninvasive imaging biomarkers for the early detection of recurrence in patients with advanced cervical cancer after radiotherapy. These parameters could help clinicians adjust therapeutic strategies and offer more personalized anticancer therapies [15].

For patients with increased PSA levels or clinical indications of disease on digital rectal examinations, transrectal ultrasound-guided biopsies are currently the most accepted definitive diagnostic method; however, biopsy results have been markedly inconsistent with the histopathology of complete prostatic resections. In this study, we used whole-lesion histogram and texture analysis to overcome this issue.

There were several limitations in this study. First, the sample size was relatively small and the diagnostic performance was not validated on an independent dataset in the current study. We will enlarge the sample size and validate it in the future study. Second, this study mainly focused on patients who had histopathological proved to be positive as well as had definitive lesions in MRI, which may introduce a selection bias for these patients. Third, the histopathologic results of most patients were obtained by transrectal ultrasound-guided biopsy; and therefore, a mismatch between the pathologic locations and delineated ROIs was present. In future studies, 3D MRI-guided biopsies might help to obtain accurate matching between MRIs and pathologic locations and improve the repeatability of prostate MR image interpretations using texture analysis results. Fourth, ROIs of the lesions were acquired on DWI images and transferred to ADC maps and T2W images. Finally, it was retrospective study and all cases came from a single center. Future studies will be prospective and have larger sample sizes. Multiple centers and MR vendors will also be assessed to confirm the current findings.

## Conclusions

In conclusion, parameters derived from whole-lesion histogram and texture analyses of ADC maps and T2W images could be used as imaging biomarkers to assess the biologic characteristics of PCa and BPH lesions, however, histogram parameters outperformed texture parameters in the diagnostic performance, which could help clinicians differentiate benign and malignant prostate nodules, providing efficient and accurate clinical decisions.

## Data Availability

The datasets used and/or analyzed during the current study are available from the corresponding author on reasonable request.

## Abbreviations

ADC: Apparent diffusion coefficient
AUC: Area under the curve
BPH: Benign prostatic hyperplasia
DWI: Diffusion-weighted imaging
FOV: Field-of-view
MRI: Magnetic resonance imaging
PCa: Prostate cancer
PSA: Prostate-specific antigen
ROC: Receiver operating characteristic
TR/TE: Repetition time/echo time
